# Interventions to promote cancer awareness and early presentation: systematic review

**DOI:** 10.1038/sj.bjc.6605388

**Published:** 2009-12-03

**Authors:** J Austoker, C Bankhead, L J L Forbes, L Atkins, F Martin, K Robb, J Wardle, A J Ramirez

**Affiliations:** 1 Cancer Research UK Primary Care Education Research Group, Cancer Epidemiology Unit, University of Oxford, Richard Doll Building, Roosevelt Drive, Oxford, UK; 2 Department of Primary Care, Oxford Centre for Monitoring and Diagnosis (MaDOx), University of Oxford, Rosemary Rue Building, Oxford, UK; 3 Kings College London, Cancer Research UK Promoting Early Presentation Group, Institute of Psychiatry, St Thomas' Hospital, London SE1 7EH, UK; 4 Department of Epidemiology and Public Health, Health Behaviour Research Centre, University College London, Gower Street, London, UK

**Keywords:** cancer awareness, cancer knowledge, delay, complex interventions, early presentation, health service utilisation

## Abstract

**Background:**

Low cancer awareness contributes to delay in presentation for cancer symptoms and may lead to delay in cancer diagnosis. The aim of this study was to review the evidence for the effectiveness of interventions to raise cancer awareness and promote early presentation in cancer to inform policy and future research.

**Methods:**

We searched bibliographic databases and reference lists for randomised controlled trials of interventions delivered to individuals, and controlled or uncontrolled studies of interventions delivered to communities.

**Results:**

We found some evidence that interventions delivered to individuals modestly increase cancer awareness in the short term and insufficient evidence that they promote early presentation. We found limited evidence that public education campaigns reduce stage at presentation of breast cancer, malignant melanoma and retinoblastoma.

**Conclusions:**

Interventions delivered to individuals may increase cancer awareness. Interventions delivered to communities may promote cancer awareness and early presentation, although the evidence is limited.

Late stage at diagnosis is a major factor accounting for survival differences between European countries for several cancers (Gatta *et al*, 2000; Sant *et al*, 2003, 2007). For some cancers, for example breast cancer, late stage at diagnosis has been shown to contribute to the difference in survival between rich and poor (Downing *et al*, 2007) and black and white women (Jack *et al*, 2009).

Patient delay in presenting for medical help after symptom discovery is likely to contribute to late stage at diagnosis. Low cancer awareness (which may include knowledge or beliefs about cancer symptoms, risk of developing cancer, risk factors, effectiveness of treatment or effectiveness of strategies for early detection) is a risk factor for patient delay (Ramirez *et al*, 1999; MacDonald *et al*, 2004).

In 2003, the Department of Health commissioned a systematic review of evidence about factors influencing delay in cancer diagnosis. While this was not its main focus, it included studies examining effectiveness of interventions to reduce patient delays in cancer diagnosis (MacDonald *et al*, 2004). It concluded that there had been little research in this area, but that public cancer awareness campaigns had been associated with some improvements in awareness and diagnosis of cancer, but that the long-term benefits were unclear.

The lack of evidence about the effectiveness of interventions to promote cancer awareness and early presentation is hampering development of policy and local action. The aim of this study was to examine the evidence of effectiveness of interventions to raise cancer awareness and promote early presentation with cancer symptoms to inform policy and future research.

## Materials and methods

### Search strategy

We searched the peer-reviewed literature published in English for studies examining the effectiveness of interventions to increase cancer awareness or promote early presentation. We searched the Cochrane Library, Medline, EMBASE and PsychINFO from 2000 to November 2008 (see Appendix A online for search strategy). Two reviewers identified relevant studies from titles and abstracts; a third reviewer resolved disagreements. We checked the reference lists of identified reports for further relevant studies.

### Study selection criteria

We included studies examining interventions in any population except those targeting only people at high genetic risk or aiming to increase cancer awareness in health professionals exclusively. We searched for studies examining effectiveness of two types of intervention:

- Interventions delivered to identified individuals recruited to a study, which attempted to collect outcome data from those individuals after the intervention; for example a one-to-one interaction with a health professional or a leaflet given or posted to an identified individual (‘individual-level interventions’).
- Interventions delivered to communities in which researchers did not control or identify which individual received the intervention, for example media campaigns, leaflets distributed indiscriminately at a health club, street stalls with posters and leaflets to promote early presentation (‘community-level interventions’).

For individual-level interventions, we searched for randomised controlled trials in which the comparator was placebo, no intervention or usual care. We excluded studies comparing two different interventions or variants of an intervention.

For community-level interventions, we searched for controlled trials (with contemporaneous controls, randomised or non-randomised, with comparator no intervention) and uncontrolled studies that collected data on outcomes before and after the intervention. This was to acknowledge that evaluating community-level interventions in randomised controlled trials is difficult and that policy on implementation of these is often made on the basis of less rigorous evaluations.

We categorised each type of study by whether the outcome related to cancer awareness or early presentation. We included studies with any one of the following cancer awareness outcomes: knowledge or beliefs about cancer symptoms, what to look for when detecting a change that might be cancer, risk of cancer, cancer risk factors, effectiveness of cancer treatment if given early, natural history or prognosis of cancer, attitudes towards early detection behaviours and help seeking or confidence to detect a change that might be cancer. We included studies with any one of the following outcomes that might reflect early presentation: time from symptom discovery to presentation or diagnosis, stage of disease at diagnosis or survival/mortality.

We excluded studies examining exclusively any of the following outcomes: knowledge of or beliefs about nature of treatment for cancer, cancer screening or checking behaviours (for example checking breasts, testicles or skin); health-checking behaviour (for example frequency of or competency in breast, testicular or skin self-examination); intentions to take up screening or screening uptake. We excluded studies with composite outcomes including the outcomes of interest, where these were not reported separately.

We also excluded studies in which the only post-intervention outcome measure was taken on the same day the intervention was delivered (see Appendix B online for summary of inclusion and exclusion criteria).

Two reviewers independently extracted data from all papers identified as potentially relevant onto a data extraction form (Appendix C online). Two reviewers independently applied the inclusion criteria and a third reviewer resolved disagreements.

### Quality assessment

The quality of randomised controlled trials eligible for inclusion in the review was scored using a methodology checklist developed previously by members of the review team (Goldsmith *et al*, 2006) (Appendix D online provides the form used by reviewers to measure quality). Each criterion on the checklist was assessed as *well covered*, *adequately addressed*, *poorly addressed*, *not reported* or *not applicable*. The methodological quality of each study was then rated as: ++ (all or most of the criteria have been fulfilled), + (some of the criteria have been fulfilled) or − (few or no criteria have been fulfilled). We did not formally score quality of studies of community-level interventions.

### Data synthesis

We conducted non-quantitative synthesis of evidence by preparing tables summarising the results of studies for each of the main outcomes of interest.

## Results

**Figure 1:**
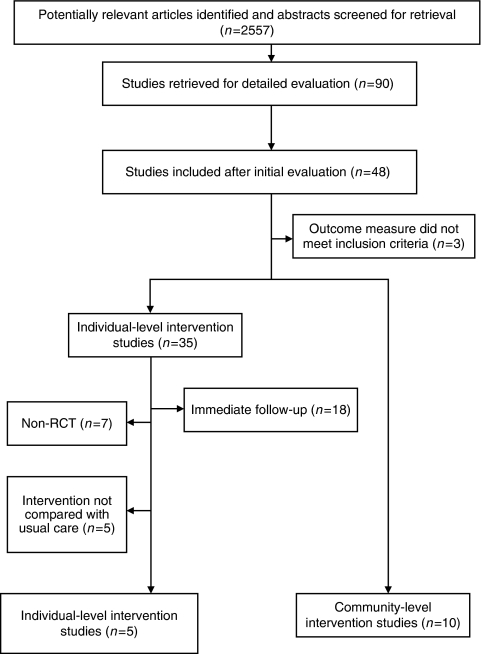
Flow of studies.

The search strategy identified 2557 abstracts. Of these, 90 were identified as meeting the criteria and we obtained full text versions of these reports. We subsequently found that 42 of these were not relevant. We excluded three out of the remaining 48 reports because the outcomes did not meet the inclusion criteria. This left 35 studies of individual-level interventions and 10 of community-level interventions. From the individual-level interventions, we excluded 18 because outcomes were measured on the same day as the delivery of the intervention, 7 because they were not randomised controlled trials and 5 because the studies compared interventions with other interventions, rather than no intervention or usual care (Figure 1).

### Individual-level interventions

We found five randomised controlled trials of individual-level interventions examining cancer awareness outcomes and none examining early presentation outcomes.

#### Description of studies and interventions

The five randomised controlled trials were carried out in the United Kingdom, United States and the Netherlands and are described in Table 1. Two were cluster randomised controlled trials (Boundouki *et al*, 2004; Glazebrook *et al*, 2006). The trials focused on all cancers (de Nooijer *et al*, 2004), prostate cancer (Wilt *et al*, 2001), breast cancer (Rimer *et al*, 2002), oral cancer (Boundouki *et al*, 2004) and malignant melanoma (Glazebrook *et al*, 2006). Four of the trials examined the effectiveness of written information compared with no written information, either sent by post (Wilt *et al*, 2001; Rimer *et al*, 2002; de Nooijer *et al*, 2004) or given out in a waiting room (Boundouki *et al*, 2004). In one trial, the written information was supplemented by telephone counselling in a third arm (Rimer *et al*, 2002). Another trial examined the additional effect of tailoring the postal information to individual knowledge and intentions in a third arm (de Nooijer *et al*, 2004). The fifth study examined the effectiveness of an interactive computer programme in general practice (Glazebrook *et al*, 2006). All trials examined knowledge outcomes, but at different times after the intervention: 2 weeks (Wilt *et al*, 2001), 3 weeks (de Nooijer *et al*, 2004), 8 weeks (Boundouki *et al*, 2004), 6 months (de Nooijer *et al*, 2004; Glazebrook *et al*, 2006) and 24 months after (Rimer *et al*, 2002). All used different measures of cancer knowledge: three used knowledge scores encompassing a range of elements of knowledge (Boundouki *et al*, 2004; de Nooijer *et al*, 2004; Glazebrook *et al*, 2006); one study examined attitudes towards paying attention to and seeking help for symptoms (de Nooijer *et al*, 2004) and two used only one or two isolated knowledge questions, among other questions relating to screening and treatment preferences (Wilt *et al*, 2001; Rimer *et al*, 2002). For one of these studies (Rimer *et al*, 2002), this is likely to be because the main aim of the intervention was to promote uptake of breast screening, and for the second, the main aim was to inform decision making about screening, rather than to promote early presentation (Wilt *et al*, 2001).

#### Quality of studies

The quality of the five trials was moderate to good. All stated that they used randomisation, although only one described how the randomisation sequence was generated (Wilt *et al*, 2001). The nature of the interventions meant that participants could not be kept blind to treatment allocation. None of the trials reported blinding of researchers to treatment allocation at the time of outcome data collection or analysis. All the studies examined baseline demographic differences between the trial arms and all examined change in knowledge or attitude score before and after the interventions except for one (Wilt *et al*, 2001), which examined outcomes only post-intervention. This may be important because there were baseline differences between the groups in this trial. All the reports reported withdrawals from the trial. The analysis was appropriate for most studies, except one cluster randomised controlled trial, which did not analyse the data using the appropriate method for this design (Boundouki *et al*, 2004). The other cluster randomised controlled trial used appropriate random effects modelling (Glazebrook *et al*, 2006).

#### Findings

The trials were heterogeneous in terms of nature of intervention, populations and outcomes measured and, therefore, we did not attempt any quantitative synthesis. All the five trials found that the intervention increased at least one aspect of cancer awareness, although the effects were fairly modest. The most intensive intervention – tailored written information with a reinforcing newsletter at 12 months plus two telephone counselling sessions – increased the proportion who gave the correct answer to a question about age-related risk by 12% compared with usual care 2 years after the written information is sent (Rimer *et al*, 2002). Less intensive interventions increased cancer awareness more modestly (an interactive computer programme increased the average melanoma knowledge score by 6% after 6 months (Glazebrook *et al*, 2006) and a leaflet increased average oral cancer knowledge score by 4% after 8 weeks (Boundouki *et al*, 2004)). A leaflet about prostate cancer increased the proportion who knew that the effectiveness of treatment in early prostate cancer is unknown by 12% after 2 weeks, but the magnitude of this difference may be at least partly due to the short follow-up (Wilt *et al*, 2001). This trial found that the leaflet did not increase knowledge of the natural history of untreated early prostate cancer.

We found some evidence that tailored print information was more effective than general information; tailored information increased average cancer knowledge scores by about 11% compared with no information and 4% compared with general information after 3 weeks (de Nooijer *et al*, 2004). Tailored print information modified attitudes towards paying attention to and seeking help for symptoms only very modestly (1–2% change in average scores) compared with no information (de Nooijer *et al*, 2004).

### Community-level interventions examining cancer awareness

#### Description of studies and interventions

We found four studies examining the effectiveness of community-level interventions aiming to increase cancer awareness (Table 2): all were controlled studies, but none used randomisation (Kiekbusch *et al*, 2000; Skinner *et al*, 2000; Blumenthal *et al*, 2005; McCullagh *et al*, 2005). The interventions were a public education campaign to increase cancer awareness in African-American communities in two US cities (Blumenthal *et al*, 2005); an educational programme to promote breast cancer awareness in African-American women in one US city (Skinner *et al*, 2000); a multimedia programme to promote malignant melanoma knowledge sited in a kiosk in a public place in a Swedish village (Kiekbusch *et al*, 2000) and a health promotion initiative to promote testicular cancer knowledge and self-checking using posters, leaflets and shower gel in UK workplaces, health clubs and leisure centres (McCullagh *et al*, 2005).

The studies used different outcome measures, one encompassing knowledge, beliefs and attitudes (Blumenthal *et al*, 2005), the others only knowledge (Kiekbusch *et al*, 2000; Skinner *et al*, 2000; McCullagh *et al*, 2005); only one used a measure that was reported to have been validated (Skinner *et al*, 2000).

#### Quality of studies

In all the studies, the researchers selected controls appropriately by identifying communities or sites that were likely to have populations with similar characteristics to the intervention communities or sites, but were not likely to be contaminated by the intervention. For two of the studies (the Swedish study of the melanoma interactive multimedia programme, Kiekbusch *et al*, 2000, and the US study of the breast cancer educational programme, Skinner *et al*, 2000), the researchers used only one control area. The public education campaign selected two control cities (Blumenthal *et al*, 2005) and the UK study of the testicular cancer initiative selected four control sites (McCullagh *et al*, 2005). While the study design in these four studies is stronger than if they were uncontrolled, differences between intervention and control areas can give rise to spurious findings of effectiveness or lack of effectiveness.

#### Findings

The studies examining the effectiveness of the public education campaign in the United States and the effectiveness of the interactive multimedia kiosk in Sweden found no effect on knowledge (Kiekbusch *et al*, 2000; Blumenthal *et al*, 2005). The studies of the educational programme for breast cancer in the United States and the UK health promotion initiative for testicular cancer found modest increases in knowledge, the first an increase in average breast cancer knowledge score of about 6% after 8 months (Skinner *et al*, 2000) and the second an increase in average testicular cancer knowledge score of 20% after 6 weeks (McCullagh *et al*, 2005).

### Community-level interventions examining early presentation outcomes

#### Description of studies and interventions

We found six studies; one interrupted time-series analysis (Catalano *et al*, 2003) and five before-and-after studies (Rossi *et al*, 2000; Geczi *et al*, 2001; MacKie *et al*, 2003; Leander *et al*, 2007; Gabram *et al*, 2008) (Table 3).

The interrupted time-series study examined the effectiveness of an annual media campaign, Breast Cancer Awareness Month, over 23 years in three US cities (Catalano *et al*, 2003). One before-and-after study examined the effectiveness of educational presentations at a range of sites aiming to downstage breast cancer at the time of diagnosis in African-American women in a US city (Gabram *et al*, 2008). The other four studies examined effectiveness of public education campaigns. Two aimed to promote early presentation in malignant melanoma: a poster and leaflet campaign in the West of Scotland (MacKie *et al*, 2003); and a media campaign followed by a leaflet to every household inviting every adult with risk factors for a skin check in one city in Italy (Rossi *et al*, 2000). One examined the effectiveness of a national testicular cancer awareness campaign in Hungary (Geczi *et al*, 2001) and another a national retinoblastoma awareness campaign in Honduras (Leander *et al*, 2007); both used broadcast and print media, and seminars and presentations to groups.

Three studies collected outcome data on time from symptom discovery to presentation or diagnosis (Geczi *et al*, 2001; MacKie *et al*, 2003; Leander *et al*, 2007). Five studies collected stage at diagnosis as an outcome (Rossi *et al*, 2000; Catalano *et al*, 2003; MacKie *et al*, 2003; Leander *et al*, 2007; Gabram *et al*, 2008).

#### Quality of studies

The time-series study was of high quality, the analysis controlling for autocorrelation, secular trends and events that might increase detection of all tumours (such as open enrolment to health insurance plans) by modelling as a function of the incidence of early stage colon cancers in men (Catalano *et al*, 2003). A before-and-after design is often the only feasible design for evaluating public education campaigns, although this design is intrinsically limited because change in outcome cannot be attributed to the intervention alone. However, in four of the before-and-after studies, the outcomes were measured soon after the intervention (Rossi *et al*, 2000; Geczi *et al*, 2001; Leander *et al*, 2007; Gabram *et al*, 2008) so changes are fairly likely to be attributable to the intervention. The Scottish melanoma study examined outcomes 10 years after the intervention (MacKie *et al*, 2003); however, a study examining earlier outcomes of the campaign suggests that the campaign immediately and significantly increased the proportion of malignant melanomas with Breslow thickness <1.5 mm and that this was sustained during the 1980s (MacKie and Hole, 1992).

#### Findings

The time-series study found that Breast Cancer Awareness Month, over 23 years, led to the detection of 790 more early stage (*in situ* and local (confined to the breast)) breast cancers (an average of 34 per year) during the quarters in which the month occurred (Catalano *et al*, 2003). The authors neither report *in situ* and local cancer separately, nor the proportion identified by screening. The study of educational presentations to downstage breast cancer in African-American women found that it reduced the proportion with advanced disease and increased the proportion with very early disease (Stage 0) (Gabram *et al*, 2008). The study of the Italian melanoma campaign found a reduction in mean tumour thickness over the period of the campaign compared with the 4 years before (Rossi *et al*, 2000), and the study of the Scottish melanoma campaign found an increase in the proportion of cases with tumour thickness <1.5 mm (MacKie *et al*, 2003). This study also found an increase in the proportion delaying presentation for <3 months. The two other studies examining time from symptom discovery to diagnosis found that the campaigns had no effect (Geczi *et al*, 2001; Leander *et al*, 2007). However, the Honduran retinoblastoma campaign was associated with a reduction in the proportion presenting with advanced disease (Leander *et al*, 2007).

## Discussion

### Summary of findings

We found limited evidence to inform policy on individual- or community-level interventions to promote cancer awareness. Randomised controlled trials of several individual-level interventions, which included written information (tailored and general), telephone counselling and a computer interactive programme, found modest positive effects on cancer knowledge or attitudes. Follow-up was for 6 months or less for all except one of the trials, so the long-term benefits are not clear. More intensive and tailored interventions are likely to be more effective. We found no evidence to inform policy on interventions delivered to individuals to promote early presentation. We found limited evidence of effectiveness of community-level interventions (small group educational programmes and health promotion programmes in workplaces, health clubs and leisure centres) to promote cancer awareness. We found good evidence that Breast Cancer Awareness Month in the United States promotes diagnosis of breast cancer at an early stage and some evidence that educational interventions by community health advocates and public education campaigns downstage breast cancer, malignant melanoma and retinoblastoma and reduce time from symptom discovery to initial presentation in melanoma. Only for the Scottish malignant melanoma campaign did we find any evidence that the effect was sustained over a number of years.

Our systematic review has identified stronger evidence for interventions to promote cancer awareness and early presentation than the previous report, which found five studies (seven reports) that would have met our inclusion criteria, had we extended our search to studies published earlier (MacDonald *et al*, 2004). Two of the reports examined earlier outcomes of the Scottish melanoma campaign that we have referred to above (Doherty and MacKie, 1988; MacKie and Hole, 1992). One study (a controlled study of a community-level intervention examining early presentation outcomes) examined the effectiveness of a cervical cancer group education intervention in rural India. The intervention increased the proportion of early cervical cancers diagnosed in the intervention area compared with neighbouring areas (Jayant *et al*, 1995). The remaining four reports examined three interventions aiming to increase malignant melanoma awareness: one individual-level intervention (an educational brochure distributed in the workplace to increase knowledge in Australian men aged 45 years and older) examined in a randomised controlled trial, which found that it increased knowledge of melanoma compared with no brochure after 3 months (Hanrahan *et al*, 1995) and two fairly small-scale UK public education campaigns, neither of which found good evidence of a reduction in tumour thickness after the campaigns, although this may have been due to small numbers of incident cancers (Whitehead *et al*, 1989; Graham-Brown *et al*, 1990; Healsmith *et al*, 1993).

### Strengths and weaknesses of the review

Our study brings together the available evidence of effectiveness of interventions to promote cancer awareness and early presentation. Our search strategy was pragmatic and aimed to be specific, but did not include the ‘grey’ literature (that not published in peer-reviewed journals). There is some evidence that more comprehensive search strategies have little effect on the overall result of systematic reviews and may introduce bias by including studies with weaker designs (Egger *et al*, 2003). However, in systematic reviews of social interventions, such as public education campaigns or health promotion initiatives, searching databases other than the standard biomedical ones may uncover important studies (Ogilvie *et al*, 2005). While we did not search other databases, we relaxed our study design inclusion criteria for evaluations of community-level interventions, recognising that controlled trials, and particularly randomised controlled trials, are more difficult to carry out.

Searching databases for studies of any kind of intervention to promote cancer awareness or early presentation is difficult because the search terms cannot focus on the intervention itself, unlike a search for studies of the effectiveness of a drug or a particular type of complex intervention. A systematic review of interventions to communicate risk also documented this difficulty (Matthews *et al*, 1999). It is possible that we missed some studies because of the difficulties of designing a search with a high level of sensitivity and specificity.

Knowledge of screening, screening uptake and self-checking behaviour – for example breast checking (including breast self-examination) or testicular checking – may be considered to be important elements of cancer awareness. We excluded studies of interventions examining only the outcomes of knowledge or uptake of breast or cervical screening because these have been covered by other studies (Bonfill Cosp *et al*, 2001; Forbes *et al*, 2002). We excluded studies examining outcomes of self-checking behaviour because the effectiveness of different modes of self-examination has not been established.

### Strengths and weaknesses of the available evidence

For interventions delivered at an individual level, we found five fairly well-conducted randomised controlled trials examining awareness outcomes. None examined early presentation outcomes. In two of the trials, only one or two relevant knowledge questions were included as outcomes (Wilt *et al*, 2001; Rimer *et al*, 2002) because the main aim of the interventions were not, primarily, to increase cancer awareness to promote early presentation, but to promote breast cancer screening in one (Rimer *et al*, 2002) and decision making about prostate cancer screening in the other (Wilt *et al*, 2001). The other three interventions did aim mainly to increase awareness to promote early presentation in malignant melanoma (Glazebrook *et al*, 2006) oral cancer (Boundouki *et al*, 2004) and a range of cancers (de Nooijer *et al*, 2004).

Cancer awareness was measured in a number of ways. Only one trial used a knowledge scale that was reported to have been validated (Boundouki *et al*, 2004). Owing to this and the short follow-up in all except one trial, it is not possible to assess whether the increases in awareness would be sufficiently comprehensive, large or sustained to lead to significant behavioural change in the event of symptom discovery.

One of the difficulties of evaluating community-level interventions using the positivist methods conventional in medical research is that these methods are less widely accepted by social science and health promotion disciplines involved in designing them (Green and Tones, 1999; Ogilvie *et al*, 2005). Another is that the interventions are usually complex (multi-component) and dependent on context, and controlled trials, let alone randomised controlled trials, are often very difficult (Thomson *et al*, 2004). We found four controlled studies (not using randomisation) of community-level interventions to increase cancer awareness. Interpretation of findings is limited by the relatively weak study design. The studies used a range of outcome measures; only one used a measure that was reported to be validated (Skinner *et al*, 2000). Two studies found no significant effects on cancer awareness (Blumenthal *et al*, 2005) (Kiekbusch *et al*, 2000); whether this is due to intrinsic lack of effectiveness of the interventions, invalid outcome measures or to limitations of study design is unknown. Two found increases in cancer awareness: one 8 months after an intensive educational programme (Skinner *et al*, 2000) and one 6 weeks after a poster and leaflet initiative (McCullagh *et al*, 2005). It is likely that the outcomes were attributable to the interventions, but we cannot be sure of this because of the limitations of study design.

Overall, community-level interventions to promote early presentation provided some evidence of effectiveness for breast cancer, melanoma and retinoblastoma. Five studies suggested that educational campaigns may lead to downstaging cancer (Rossi *et al*, 2000; Catalano *et al*, 2003; MacKie *et al*, 2003; Leander *et al*, 2007; Gabram *et al*, 2008); however, all were uncontrolled, so the results cannot be reliably attributed to the intervention. On the other hand, outcomes were measured soon after the intervention, so it is more likely that the improvement can be attributed to it. Another problem with interpreting the findings is that it is not possible to attribute the downstaging of cancer to the effect of the campaigns on the public only – all the interventions are likely to have raised health professional awareness as well; in fact, most were specifically designed to do so.

The finding that Breast Cancer Awareness Month (Catalano *et al*, 2003) increased diagnosis of early stage tumours may be at least partly due to increased mammography uptake during the month or soon after, rather than early presentation with symptoms, so we cannot tell which kind of behaviour was promoted by the intervention. This is also true of the finding that educational presentations increased the proportion with stage 0 breast cancer (Gabram *et al*, 2008). The benefit of detecting more stage 0/*in situ* cancers in terms of breast cancer outcomes is unknown, as some of the women with these cancers may never have experienced clinical problems and may have received unnecessary investigations.

Few studies examined duration of symptoms from discovery to initial presentation (Geczi *et al*, 2001; MacKie *et al*, 2003; Leander *et al*, 2007) and two found no effect (Geczi *et al*, 2001; Leander *et al*, 2007). It is possible that these two studies found no effect on duration of symptoms because the campaigns may have advanced both the average date of symptom discovery and the average date of presentation, which would lead to presentation at an earlier stage, but would have no effect on duration of symptoms.

### Implications

We found some evidence that interventions delivered at an individual level can promote cancer awareness over the short term, but no evidence that these promote early presentation with cancer symptoms. Future research evaluating individual-level interventions to promote cancer awareness should attempt to use study designs that generate high-quality evidence, measure outcomes over a longer term (months/years) and attempt to measure behavioural and stage outcomes, as well as knowledge and attitudes. We also highlight the need for standardised and validated measures of cancer awareness for different cancers, similar to the Cancer Research UK Cancer Awareness Measure supported by the National Awareness and Early Diagnosis Initiative (Stubbings *et al*, 2009). There is also a need for standardised and validated measures of duration of symptoms.

We found limited evidence that intensive education campaigns may lead to greater cancer awareness and earlier presentation over the short term. However, what exactly a campaign needs to include to make it work, to make it work over the longer term and in different settings and to make it work cost-effectively are not clear and warrant more research.

## Conflict of interest

The authors declare no conflict of interest.

## Figures and Tables

**Table 1:** Studies examining the effectiveness of individual-level interventions

**Reference**	**Cancer**	**Design**	**Intervention**	**Participants**	**Outcome (time of measurement)**	**Results**	**Quality of evidence (see Appendix D online)**
de Nooijer *et al* (2004)	Any cancer	RCT comparing: Individually tailored information *vs* General information *vs* No information	Tailored information delivered by post: letter tailored to individual based on knowledge and intentions. Included information on cancer symptoms (for several cancers), reasons for early detection, risk, breast and testicular self-examination, screening programmes. General information delivered by post: brochure on early detection in several cancers used by Dutch Cancer Society.	1331 adults (mean age 47, 80% women) without cancer recruited through newspaper adverts in the Netherlands	Knowledge of cancer symptoms (range 0, 15) (3 weeks) Attitude towards paying attention to symptoms (range −3, 3) (6 months) Attitude towards seeking help for symptoms (range −3, 3) (6 months)	Higher in tailored information group *vs* general information group *vs* control (9.85 *vs* 9.26 *vs* 8.21, *P*<0.001) Higher in tailored information group *vs* general information group *vs* control (2.05 *vs* 2.05 *vs* 1.96, *P*<0.01) Higher in tailored information group *vs* general information group *vs* control (2.13 *vs* 2.09 *vs* 1.99, *P*<0.001)	+
Rimer *et al* (2002)	Breast	RCT comparing: Tailored print materials *vs* Tailored print materials plus telephone Counselling *vs* Usual care	Tailored information delivered by post: booklet about breast cancer risk, risk factors and mammography tailored to individual based on responses provided during telephone call. Reinforcing newsletter 12 months later. Tailored information plus telephone counselling: As above plus two telephone calls (one after booklet and one after newsletter) from trained health advisor asking questions about booklet/newsletter content to elicit questions and concerns.	1091 women (aged 42–57) enrolled in health insurance plan in United States	Knowledge that women aged >50 at higher risk of breast cancer than younger women (24 months)	Higher in tailored print materials plus telephone counselling group *vs* tailored print materials group *vs* usual care (32% *vs* 26% *vs* 20%, *P*=0.001)	+
Glazebrook *et al* (2006)	Melanoma	Cluster RCT (unit of randomisation=practice) comparing: Educational *vs* No programme	Computer-based interactive educational programme to increase melanoma knowledge (including risk of sun exposure, how to protect skin, early signs) accessed through dedicated workstation in GP practice.	589 adults (mean age 38, 80% women) recruited from people with 1+ risk factor for melanoma attending general practice in United Kingdom	Knowledge of how to reduce risk of melanoma, risk factors, symptoms (range 0, 12) (6 months)	Higher in programme group *vs* no programme group (4.12 *vs* 3.36, *P*<0.001)	
Boundouki *et al* (2004)	Oral	Cluster RCT (unit of randomisation=session) comparing: Leaflet *vs* No leaflet	Leaflet to increase knowledge of oral cancer signs, risk factors and how to detect oral cancer, given out in waiting room.	316 adults (mean age 47, 59% women) attending dentist in United Kingdom	Knowledge of oral cancer (range 0, 36) (8 weeks)	Higher in leaflet group *vs* no leaflet group (30.3 *vs* 29.0, *P*<0.001)	+
Wilt *et al* (2001)	Prostate	RCT comparing: Leaflet *vs* No leaflet	Leaflet to increase knowledge about risks and benefits of early prostate cancer detection and treatment delivered by post.	550 men (mean age 72) attending a primary care centre in United States	Knowledge of natural history of untreated early prostate cancer (2 weeks) Knowledge that effectiveness of treatment in early prostate cancer is unknown (2 weeks)	No difference Higher in leaflet group *vs* no leaflet group (56% *vs* 44%, *P*=0.04)	+

Abbreviations: GP=general practitioner; RCT=randomised controlled trial.

**Table 2:** Studies examining the effectiveness of community-level interventions on cancer awareness outcomes

**Reference**	**Cancer**	**Design**	**Intervention**	**Population providing outcome data**	**Outcome**	**Results**
Blumenthal *et al* (2005)	Any cancer	Controlled study (non-randomised) comparing: Areas with black population in Nashville and Atlanta *vs* Areas with black population in two cities receiving no campaign	Public education campaign in two US cities (Nashville and Atlanta) to increase knowledge of several cancers in African-American communities, delivered by broadcast and print media, lectures, workshops, lectures, presentations over 18 months in 1994–1996.	African-American adults living in the four cities approached by random digit dialling (4053 before intervention; 3914 after intervention)	Knowledge, beliefs and attitudes towards cancer risk factors and screening	No difference. Quantitative data not provided.
Skinner *et al* (2000)	Breast	Controlled study (non-randomised) in one US city (St Louis) comparing: one managed social network for low income elderly people receiving the programme *vs* one similar managed social network not receiving the programme	Educational programme delivered in small groups by a health professional to 32 women (mainly African-American) over three sessions, to increase breast cancer knowledge and screening uptake and promoting message dissemination to others in the social network.	153 women (mean age 73) 99% African-American, members of the social network provided data both before-and-after intervention	Knowledge of breast cancer symptoms, risk factors and risk (range 0, 8) after 8 months	Higher in group education programme *vs* control networks 4.1 *vs* 3.6, *P*<0.0001)
Kiekbusch *et al* (2000)	Melanoma	Controlled study (non-randomised) in Sweden comparing: one village receiving kiosk *vs* one similar village not receiving kiosk	Interactive multimedia programme housed in kiosk in the centre of a village (in the pharmacy, then health centre, then library) to increase melanoma knowledge over 3 years.	Swedish adults aged 20–59 living in the villages recruited from population registries (648 before intervention; 604 after intervention)	Knowledge of melanoma symptoms, risk factors, risk, preventive measures (range 1, 3) at the end of intervention	No difference (kiosk village *vs* control village: Men: 2.70 *vs* 2.68, *P*-value not provided; women: 2.72 *vs* 2.75, *P*-value not provided)
McCullagh *et al* (2005)	Testicular	Controlled study (non-randomised) in the United Kingdom comparing: ten sites receiving the health promotion initiative *vs* four sites receiving no health promotion initiative	Health promotion initiative with printed shower gel sachets, stickers and posters displayed in changing rooms in workplaces, health clubs and leisure centres, to increase knowledge of testicular cancer and promote self-examination, delivered once to each site.	Men aged 15–44 attending workplaces, health clubs and leisure centres in United Kingdom (518 before intervention; 356 after intervention)	Knowledge of testicular cancer symptoms, risk and survival (range 0, 5) after 6 weeks	Higher in health promotion initiative sites *vs* control sites (4 *vs* 3, *P*=0.014)

**Table 3:** Studies examining the effectiveness of community-level interventions on early presentation outcomes

**Reference**	**Cancer**	**Design**	**Intervention**	**Population providing outcome data**	**Outcome**	**Results**
Catalano *et al* (2003)	Breast	Interrupted time-series analysis	22 annual public education broadcast and print media campaigns in three US cities (Atlanta, Detroit and San Francisco) about nature, detection and treatment of breast cancer (Breast Cancer Awareness Month) over 1975–97.	All cancer registrations in Atlanta, Detroit, San Francisco over 23 years	Additional *in situ* and local breast cancers	790 additional cancers over 23 years (*P*<0.05)
Gabram *et al* (2008)	Breast	Before-and-after study	Educational presentations delivered to groups (mainly African-American) by community health advocates in churches, workplaces, schools, etc, in one US city (Atlanta) to reduce breast cancer stage at presentation, during 2001–2004.	Women diagnosed with breast cancer (89% African-American) in one Atlanta hospital in 2001 (*n*=113) and 2004 (*n*=128)	Proportion with stage 0 Proportion with stage IV	Increased (12% *vs* 26%, *P*<0.005) Reduced (17% *vs* 9%, *P*<0.05)
MacKie *et al* (2003)	Melanoma	Before-and-after study	Public education campaign in West of Scotland to encourage early presentation in melanoma, delivered by posters and leaflets during 1986–1988.	Scottish people diagnosed with melanoma in one Glasgow clinic in 1986 (*n*=125) and 2001 (*n*=162)	Proportion delaying presentation after symptom discovery three or fewer months	Increased (16% *vs* 67%, 95% confidence interval for difference 42% to 61%)
					Proportion with tumour thickness <1.5 mm	Increased (38% *vs* 72%, 95% confidence interval for difference 23% to 45%)
Rossi *et al* (2000)	Melanoma	Before-and-after study	Public education campaign in Padua, Italy with broadcast and print media campaign followed by leaflet about symptoms and risk factors for melanoma and skin self-examination, inviting adults to request skin check, delivered by post to every family in Padua over 1991–6.	Padua residents diagnosed with melanoma between 1987–1990 (*n*=79) and 1991–1996 (*n*=137)	Mean tumour thickness	Reduced (2.0 mm *vs* 1.5 mm, *P*<0.02)
Geczi *et al* (2001)	Testicular	Before-and-after study	National Hungarian public education campaign about risk factors, importance of early detection and self-examination in testicular cancer, delivered by broadcast and print media and at events over 1995–1998.	Hungarian men diagnosed with testicular cancer in 1994 (*n*=230) and 1998 (*n*=214)	Time from symptom discovery to diagnosis	No change
Leander *et al* (2007)	Retinoblastoma	Before-and-after study	National Honduran public education campaign to increase awareness of early signs of retinoblastoma and to encourage early presentation, delivered by flyers, posters, broadcast and print media and seminars during 2003–2005.	Honduran children diagnosed with retinoblastoma in 1995–2003 (*n*=59) and 2003–2005 (*n*=23)	Proportion presenting with advanced disease Time from symptom discovery to diagnosis	Reduced (73% *vs* 35%, *P*=0.002) No change
