# Supplementary material for: Interventions to promote cancer awareness and early presentation: systematic review
Source: Br J Cancer. 2009 Dec 3;101(Suppl 2):S31–9. doi: 10.1038/sj.bjc.6605388 (PMC2790702; doi:10.1038/sj.bjc.6605388)
Supplement: Supplementary Information [file 6605388x1.doc]

**Appendix A: Search Strategy**

| Search number | Search Terms | Results |
| --- | --- | --- |
| 1 | (delay* or late or later or early or earlier or postpone* or wait* or deny or denial or promot*).mp (mp=ti,ot,ab,nm,hw,tc,id,sh,tn,dm,mf) | 3 471 505 |
| 2 | (helpseeking or diagnos* or present* or detect* or present* or attend* or consult* or seek or sought or refer or treatment or care).mp | 12 653 832 |
| 3 | (symptom* and (detect* or duration or onset*)).mp | 233 071 |
| 4 | (neoplasm* or cancer* or tumor or tumour or malignan*).mp | 3 530 224 |
| 5 | ((1 and 2) or 3) and 4 | 397 046 |
| 6 | Health education.kf,sh,kw,id or “patient education as Topic”/ | 132 915 |
| 7 | 6 and 4 | 11 986 |
| 8 | 7 or 5 | 406 275 |
| 9 | Limit 8 to yr =”2000-2008” | 217 118 |
| 10 | Limit 9 to human *(limit not valid in PsychInfo; records were retained)* | 195 231 |
| 11 | (aware* or knowledge* or attitude* or recogni* or lay concept* or health belief* or expectation or information* or education*).mp | 3 652 313 |
| 12 | Randomized Controlled Trial/ or randomized controlled trial*.mp or randomised controlled trial.mp or controlled trial*.mp or intervention.mp or Intervention Studies/ or Research Design/ or comparative study.mp or program evaluation.mp or campaign.mp or educational program*.mp or (before and after).mp or controlled study.mp | 5 908 672 |
| 13 | 10 and 11 and 12 | 11 017 |
| 14 | Immunohistochemistry.mp or stroke.mp or exp nursing staff/ or exp medical errors/ or exp malpractice/ or exp liability, legal/ or exp disease models,animal/ or exp models, biological/ or models, animal/ or ((exp RNA, neoplasm/ or exp RNA, messenger/ or exp sequence analysis, RNA/ or exp RNA/ or exp signal transduction/ or transforming growth factor beta/ or exp DNA fragmentation/ or exp apoptosis/ or exp adenoviridae/ or exp genes/ or exp gene expression/ or exp cell communication/ pr exp antigens/ or exp alternative splicing/ or exp MicroRNAs/ or gene expression/ or exp membrane proteins/ or exp DNA-binding proteins/ or intracellular signalling peptides.mp) and proteins.mp) or exp protein isoforms/ or exp proto-oncogene proteins/ or exp sequence analysis/ or exp glycosolation/ or exp chemistry, pharmaceutical/ or exp drug carriers/ or exp drug resistance/ or exp antineoplastic agents/ or exp toxicity tests/ or exp radiation oncology/ or exp cell transformation, neoplastic/ or exp mammary neoplasms, experimental/ or exp tumor stem cells/ or exp pathology/ or exp therapeutics, ae, mo, cl, nu, ct, px, ec, st, es, sn, hi, td, is, ut, mt, ve or (pa or ge or ch or ai or ut or ec or mjo or dt or pp or et or og or ah or du or im or su of tu or re or th or ad or is).fs | 16 595 285 |
| 15 | 13 not 14 | 3 103 |
| 16 | Remove duplicates from 15 | 2 759 |
| 17 | Limit 16 to English language | 2 561 |
|  | Further duplicates found and removed | 2 557 |
|  | Included at abstract stage | 90 |
|  | Included at full text stage | 48 |

**Appendix B**: **Inclusion/exclusion criteria**

**Individual level interventions**: interventions delivered to identified individuals recruited to a study which attempted to collect outcome data from those individuals after the intervention.

| Design: | RCT |
| --- | --- |
| Population: | Any, except if include only people at high genetic risk, health professionals only |
| Intervention: | Individual level intervention aimed at increasing cancer awareness or early presentation |
| Comparator: | Usual care, no intervention, placebo |
| Outcomes: | Knowledge or beliefs about:   - cancer symptoms - risk of cancer - cancer risk factors - effectiveness of early treatment for cancer - natural history or prognosis of cancer - what to look for in detecting a change that might be cancer - attitude towards early detection behaviours and help-seeking - confidence to detect a change that might be cancer   Time from symptom discovery to presentation or diagnosis; stage of disease at diagnosis; survival/mortality.  We excluded studies examining exclusively any of the following outcomes:   - knowledge of checking behaviour techniques (for example, how to check breasts, testicles or skin) - health-checking behaviour (for example, frequency of or competency in breast, testicular or skin self-examination) - knowledge of screening - knowledge of or beliefs about nature of treatment for cancer - intentions to take up screening - screening uptake   We excluded studies with composite outcomes including the outcomes of interest, where these were not reported separately.  We also excluded studies where the only post-intervention outcome measure was taken on the same day the intervention was delivered. |

**Community-level intervention:** in which researchers did not control or identify which individual received the intervention

| Design: | RCT  Controlled study not using randomisation  Before and after studies  Time-series |
| --- | --- |
| Population: | Any, except if include only people at high genetic risk, health professionals only |
| Intervention: | Community-level intervention aimed at increasing cancer awareness or early presentation |
| Comparator: | Usual care, no intervention, placebo |
| Outcomes: | Knowledge or beliefs about:   - cancer symptoms - risk of cancer - cancer risk factors - effectiveness of early treatment for cancer - natural history or prognosis of cancer - what to look for in detecting a change that might be cancer - attitude towards early detection behaviours and help-seeking - confidence to detect a change that might be cancer   Time from symptom discovery to presentation or diagnosis; stage of disease at diagnosis; survival/mortality.  We excluded studies examining exclusively any of the following outcomes:   - knowledge of checking behaviour techniques (for example, how to check breasts, testicles or skin) - health-checking behaviour (for example, frequency of or competency in breast, testicular or skin self-examination) - knowledge of screening - knowledge of or beliefs about nature of treatment for cancer - intentions to take up screening - screening uptake   We excluded studies with composite outcomes including the outcomes of interest, where these were not reported separately.  We also excluded studies where the only post-intervention outcome measure was taken on the same day the intervention was delivered. |

# Appendix C: Data Extraction Form

| **1** | **Study ID** |  |
| --- | --- | --- |
| 1.1 | ID number (reference manager ID) |  |
| 1.2 | Reference |  |
| 1.3 | 1st reviewer |  |
| 1.4 | Date of 1st review |  |
| 1.5 | 2nd reviewer |  |
| 1.6 | Date of 2nd review |  |
| **2** | **STUDY** |  |
| 2.1 | Design |  |
| 2.2 | Cancer site |  |
| 2.3 | Method of participant selection |  |
| 2.4 | Unit of randomisation |  |
| 2.5 | Specific population |  |
| 2.6 | Relevant data (i.e. the outcomes we are interested in - either cancer awareness or early presentation) |  |
| 2.7 | Objectives of intervention and paper |  |
| 2.8 | Summary of intervention, outcome measures and findings |  |
| **3** | **PARTICIPANTS** |  |
| 3.1 | Country |  |
| 3.2 | N= |  |
| 3.3 | Age |  |
| 3.4 | Gender |  |
| 3.5 | Ethnicity |  |
| 3.6 | Marital status |  |
| 3.7 | Education |  |
| 3.8 | Experience of cancer |  |
| 3.9 | Recruitment rate |  |
| 3.10. | Attrition rate |  |
| 3.11 | Income |  |
| 3.12 | Other demog. Info |  |
| **4** | **METHODS** |  |
| 4.1 | Duration of study |  |
| 4.2 | Theoretical basis of intervention |  |
| 4.3 | Type of intervention |  |
| 4.4 | Follow-up duration (time between intervention and follow-up) |  |
| 4.5 | Time points for evaluation? |  |
| 4.6 | Who delivers the intervention? |  |
| 4.7 | How is intervention delivered? |  |
| 4.8 | How many times is the intervention delivered? |  |
| 4.9 | Which outcomes have been measured? |  |
| 4.10. | How are outcomes assessed? |  |
| 4.11 | If composite score, is it possible to extract relevant data? |  |
| 4.12 | Validated measure (reference) |  |
| 4.13 | Details of measure |  |
| **5** | **STATISTICAL ANALYSIS** |  |
| 5.1 | Statistical methods used |  |
| **6** | **RESULTS** |  |
| 6.1 | Knowledge of cancer symptoms |  |
| 6.2 | Knowledge of risk factors |  |
| 6.3 | Knowledge of cancer incidence |  |
| 6.4 | Knowledge of screening availability and purpose |  |
| 6.5 | Time from symptom discovery to presentation |  |
| 6.6 | Time from symptom discovery to diagnosis |  |
| 6.7 | Size of tumour at diagnosis |  |
| 6.8 | Grade of tumour diagnosis |  |
| 6.9 | Survival |  |
| 6.10. | Differences in outcomes by age/gender/ethnicity/income/education/other |  |
| 6.11 | Other relevant observations and conclusions |  |

# Appendix D: Checklist for methodological quality of randomised studies

| 1.1 | The study addresses an appropriate and clearly focused question | Well covered  Adequately addressed  Poorly addressed | Not reported  Not applicable |
| --- | --- | --- | --- |
| 1.2 | The assignment of subjects to treatment groups is randomised | Well covered  Adequately addressed  Poorly addressed | Not reported  Not applicable |
| 1.3 | An adequate concealment method is used | Well covered  Adequately addressed  Poorly addressed | Not reported  Not applicable |
| 1.4 | Subjects and investigators are kept ‘blind’ about treatment allocation | Well covered  Adequately addressed  Poorly addressed | Not reported  Not applicable |
| 1.5 | The treatment and control groups are similar at the start of the trial | Well covered  Adequately addressed  Poorly addressed | Not reported  Not applicable |
| 1.6 | The only difference between groups is the intervention under investigation | Well covered  Adequately addressed  Poorly addressed | Not reported  Not applicable |
| 1.7 | All relevant outcomes are measured in a standard, valid and reliable way | Well covered  Adequately addressed  Poorly addressed | Not reported  Not applicable |
| 1.8 | What percentage of the individuals or clusters recruited into each treatment arm of the study dropped out before the study was completed? |  |  |
| 1.9 | All the subjects are analysed in the groups to which they were randomly allocated (often referred to as intention to treat analysis) | Well covered  Adequately addressed  Poorly addressed | Not reported  Not applicable |
| 1.10 | Where the study is carried out at more than one site, results are comparable for all sites | Well covered  Adequately addressed  Poorly addressed | Not reported  Not applicable |
| 1.11 | An appropriate analysis was used for cluster randomised controlled trials | Well covered  Adequately addressed  Poorly addressed | Not reported  Not applicable |

The methodological quality of the study is rated based on your responses to the appropriate methodology checklist using the following coding system:

| ++ | **All or most** of the criteria have been fulfilled. Where they have not been fulfilled the conclusions of the study are thought very unlikely to alter. |
| --- | --- |
| + | **Some** of the criteria have been fulfilled. Those criteria that have not been fulfilled or not adequately described are thought unlikely to alter the conclusions. |
| - | **Few or no** criteria fulfilled. The conclusions of the study are thought likely or very likely to alter. |

**Notes:**

- 1. Unless a clear and well defined question is specified, it will be difficult to assess how well the study has met its objectives or how relevant it is to the question you are trying to answer on the basis of its conclusions. Consider if the question is ‘focused’ in terms of the population studied, the intervention given and the outcomes chosen.
  2. Random allocation of patients to receive one or other of the treatments under investigation, or to receive either treatment or placebo, is fundamental to this type of study. If the description of randomisation is poor, the study should be given a lower quality rating. Consider the following points: whether the randomisation process was truly random, whether the method of allocation was described (stratification used to balance randomisation?), how the randomisation schedule was generated, how a participant was allocated to a study group and if there were any differences reported that might have explained any outcome(s) (confounding).
  3. Allocation concealment refers to the process used to ensure that researchers are unaware which group patients are being allocated to at the time they enter the study. If the method of concealment used is regarded as poor, or relatively easy to subvert, the study should be given a lower quality rating.
  4. Blinding refers to the process whereby people are kept unaware of which treatment an individual patient has been receiving when they are assessing the outcome for that patient. The higher the level of blinding, the lower the risk of bias in the study. Consider the following points: the fact that blinding is not always possible, whether every effort was made to achieve blinding and ‘observer bias’.
  5. Participants selected for inclusion in a trial must be as similar as possible. The study should report any significant differences in the composition of the study groups in relation to gender mix, age, stage of disease (if appropriate), social background, ethnic origin, or comorbid conditions. These factors may be covered by inclusion or exclusion criteria, rather than being reported directly. Failure to address this question, or the use of inappropriate groups, should lead to the study being downgraded.
  6. If some patients received additional intervention, even if of a minor nature or consisting of advice and counselling rather than a physical intervention, this treatment is a potential confounding factor that may invalidate the results. If groups were not treated equally, the study should be rejected unless no other evidence is available (if used as evidence it should be treated with caution).
  7. The primary outcome measures used should be clearly stated in the study. Where outcome measures require any degree of subjectivity, some evidence should be provided that the measures used are reliable and have been validated prior to their use in the study. Considered whether participant outcomes were reviewed at the same time intervals and if they received the same amount of attention from researchers and health workers (any differences may introduce performance bias).
  8. The number of participants that drop out of a study should give concern if the number is very high. Conventionally, a 20% drop out rate is regarded as acceptable, but this may vary. Some regard should be paid to why participants dropped out, as well as how many. It should be noted that the drop out rate might be expected to be higher in studies conducted over a long period of time. A higher drop out rate will normally lead to downgrading, rather than rejection of a study.
  9. It is rarely the case that all participants allocated to the intervention group receive the intervention throughout the trial, or that all those in the comparison group do not. However, participant outcomes must be analysed according to the group to which they were originally allocated irrespective of the intervention that they actually received (intention-to-treat analysis). The study may be rejected if it is clear that an intention-to-treat analysis was not used.
  10. In multi-site studies, confidence in the results should be increased if it can be shown that similar results were obtained at the different participating centres.
  11. The analysis chosen for cluster randomised controlled trials should be consistent with the design – it should take clustering into account. Valid approaches include: analysing clustered outcome data (unit of analysis is the same as that of randomisation) and individual level analysis accounting for clustering such as Random Effects Regression, Generalised Estimating Equations or Robust Standard Error.
